# Novel functional roles for *PERIANTHIA* and *SEUSS* during floral organ identity specification, floral meristem termination, and gynoecial development

**DOI:** 10.3389/fpls.2014.00130

**Published:** 2014-04-07

**Authors:** April N. Wynn, Andrew A. Seaman, Ashley L. Jones, Robert G. Franks

**Affiliations:** ^1^Department of Plant and Microbial Biology, North Carolina State UniversityRaleigh, NC, USA; ^2^Department of Biology, Saint Mary's College of MarylandSt. Mary's City, MD, USA; ^3^Department of Plant Biology, University of TexasAustin, TX, USA

**Keywords:** ovule, gynoecium, flowers, agamous, wuschel, organ identity, indeterminate growth

## Abstract

The gynoecium is the female reproductive structure of angiosperm flowers. In *Arabidopsis thaliana* the gynoecium is composed of two carpels that are fused into a tube-like structure. As the gynoecial primordium arises from the floral meristem, a specialized meristematic structure, the carpel margin meristem (CMM), develops from portions of the medial gynoecial domain. The CMM is critical for reproductive competence because it gives rise to the ovules, the precursors of the seeds. Here we report a functional role for the transcription factor *PERIANTHIA* (*PAN*) in the development of the gynoecial medial domain and the formation of ovule primordia. This function of *PAN* is revealed in *pan aintegumenta (ant)* as well as *seuss (seu) pan* double mutants that form reduced numbers of ovules. Previously, *PAN* was identified as a regulator of perianth organ number and as a direct activator of *AGAMOUS (AG)* expression in floral whorl four. However, the *seu pan* double mutants display enhanced ectopic *AG* expression in developing sepals and the partial transformation of sepals to petals indicating a novel role for *PAN* in the repression of *AG* in floral whorl one. These results indicate that *PAN* functions as an activator or repressor of *AG* expression in a whorl-specific fashion. The *seu pan* double mutants also display enhanced floral indeterminacy, resulting in the formation of “fifth whorl” structures and disruption of *WUSCHEL* (*WUS*) expression patterns revealing a novel role for *SEU* in floral meristem termination.

## Introduction

In *Arabidopsis thaliana*, as with most angiosperms, reproductive competence depends on the proper development of the flower. Arabidopsis flowers develop from floral meristems, specialized structures that contain organized groups of undifferentiated cells that give rise to the four types of floral organs: sepals, petals, stamens, and carpels (Vaughn, 1955; Hill and Lord, 1988; Bowman et al., 1989). These four organ types develop in concentrically-organized circular fields, termed whorls. Each organ type is produced in a separate whorl of the flower: four sepals in the exterior-most whorl (whorl 1); four petals (whorl 2); six stamens (whorl 3); and finally two carpels in the inner-most whorl (whorl 4).

The proper development of the complete complement of 16 floral organs requires a balance within the floral meristem between the generation of floral organ primordia and the maintenance or renewal of undifferentiated stem cells (Sablowski, 2007). After the initiation of the two carpels in the innermost whorl, the floral meristem terminates. Thus, the ability of the floral meristem to produce cells that will become floral organs is temporally-limited and is genetically-predetermined. This type of developmental floral program is termed a determinate program and generates a fixed number of floral organs. A failure to terminate the floral meristem can result in indeterminacy, or the formation of additional (supernumerary) organs in the central-most positions of the flower.

### *AGAMOUS* specifies stamen and carpel identity and brings about termination of the floral meristem

*AGAMOUS (AG)* encodes a MADS-domain containing protein that plays at least two key roles during floral genesis: participation in regulatory complexes that specify the identity of stamens and carpels; and bringing about termination of the floral meristem and thus generating a determinant floral structure (Yanofsky et al., 1990; Bowman et al., 1991, 2012; Coen and Meyerowitz, 1991; Drews et al., 1991; Meyerowitz et al., 1991). Loss of *AG* activity results in indeterminate flowers within which additional whorls of floral organs are generated from perdurant meristematic cells.

*AG* is predominately expressed in the inner-most two whorls of the flower (whorls 3 and 4) in the cells that will give rise to the stamens and carpels (Yanofsky et al., 1990). At least three genes have been shown to have a role in the activation of *AG* transcription within the flower: *LEAFY* (*LFY*) (Weigel and Meyerowitz, 1993); *WUSCHEL* (*WUS*) (Lenhard et al., 2001) and *PERIANTHIA* (*PAN*) (Das et al., 2009; Maier et al., 2009). All three directly bind to cis-regulatory elements located in the *AG* second intron (Busch et al., 1999; Lohmann et al., 2001; Das et al., 2009; Maier et al., 2009). *pan* single mutant plants exhibited an incompletely penetrant floral meristem indeterminacy phenotype (Das et al., 2009) and the penetrance and severity of *pan* phenotypes are modified by environmental growth conditions, particularly day length (Maier et al., 2009). Additionally *pan* mutant phenotypes are enhanced by hypomorphic *lfy* alleles, indicating a functional similarity between *LFY* and *PAN* during the activation of *AG* (Das et al., 2009).

### Repression of *AG* in whorls one and two

A number of genes have been shown to play a role in the repression of *AG* within floral whorls one and two (for review see Liu and Mara, 2010). One of these genes, *SEUSS (SEU)*, encodes a transcriptional adaptor protein, that physically interacts with several MADS domain proteins including APETALA1 (AP1), SEPALLATA3 (SEP3), AGAMOUS-LIKE24 (AGL24) and SHORT VEGETATIVE PHASE (SVP) (Sridhar et al., 2004; Gregis et al., 2006; Sridhar et al., 2006; Gregis et al., 2009). As a transcriptional adaptor, SEU is not thought to bind DNA directly but rather is recruited to cis-regulatory elements located within the *AG* second intron through interactions with these MADS domain containing DNA transcriptional regulators (Liu and Meyerowitz, 1995; Franks et al., 2002; Gregis et al., 2006; Sridhar et al., 2006). SEU functions as a bridging protein that recruits the transcriptional repressor LEUNIG (LUG) to the complex and brings about transcriptional repression of *AG* in whorls one and two (Sridhar et al., 2004, 2006). *seu* mutants display weak homeotic transformations of perianth organs caused by ectopic expression of *AG* in the perianth, as well as a variety of additional pleiotropic phenotypes (Franks et al., 2002). *SEU* is widely expressed within the developing plant and likely functions in many developmental events.

### *SEU* and *ANT* function during the development of the gynoecial medial domain

In *Arabidopsis thaliana* the female reproductive floral structure is the gynoecium, a composite structure formed from the congenital fusion of two carpel organs into a tube-like structure (Bowman et al., 1999). A specialized meristematic tissue termed the carpel margin meristem (CMM) develops within the medial portions of the gynoecial tube and gives rise to ovules (Bowman et al., 1999; Liu et al., 2000; Azhakanandam et al., 2008). The ovules are the immature, prefertilized precursors of seeds. Many groups have contributed to the understanding of the molecular mechanisms that support the specification and development of the medial gynoecial domain and the subsequent initiation of ovules (reviewed in Reyes-Olalde et al., 2013), but our mechanistic understanding of this important developmental process is incomplete.

*SEU*, in addition to its function in the specification of floral organ identity through the repression of *AG*, functions to promote ovule formation in the CMM (Azhakanandam et al., 2008). *SEU* works in a partially redundant manner with *AINTEGUMENTA* (*ANT*), another transcription factor, to regulate the expression of downstream genes critical for the formation of ovules (Azhakanandam et al., 2008; Wynn et al., 2011). In contrast to *SEU* which does not have a DNA binding domain (Sridhar et al., 2006), *ANT* encodes an *AP2-*like transcription factor containing a sequence-specific DNA binding domain. *ANT* activity supports the establishment of proper organ size in lateral organs by controlling the period of developmental time during which cells of the organ primordia are competent to grow and divide (Elliott et al., 1996; Klucher et al., 1996; Mizukami and Fischer, 2000; Nole-Wilson and Krizek, 2000; Krizek and Eaddy, 2012). The loss of either *SEU* or *ANT* activity, individually, results in a reduction of ovule number, however, the combined loss of *SEU* and *ANT* activity results in the complete loss of ovule formation (Azhakanandam et al., 2008).

Although both *SEU* and *ANT* function in *AG* repression, it is unlikely that the alteration of CMM development in the *seu ant* double mutant is due to the de-repression of *AG* expression (Azhakanandam et al., 2008). Rather additional gene regulatory alterations in the *seu ant* double mutants are likely to engender the altered development of the medial domain. Published transcriptomics experiments have identified genes that are misregulated in the *seu ant* gynoecia relative to the single mutant parents (Wynn et al., 2011). Many of these genes are expressed within the developing medial gynoecial domain and thus are likely candidates for regulators of medial domain development. *PAN* encodes one such candidate. *PAN* is a member of the bZIP transcription factor super-family of proteins (Hurst, 1995; Chuang et al., 1999). *pan* mutants display alterations in the spacing, position, and number of perianth organs formed, but do not condition a severe gynoecial phenotype (Running and Meyerowitz, 1996; Meyerowitz, 1997; Roe et al., 1997; Chuang et al., 1999; Maier et al., 2009, 2011; Wynn et al., 2011). As *PAN* is expressed strongly in the developing gynoecial medial domain, placenta, and ovules is it possible that *PAN* plays a functional role during gynoecial development that is not observed in the *pan* single mutant due to functional redundancy.

In order to better assay the functional role of *PAN* during gynoecial development, we have generated *seu pan* and *pan ant* double mutant plants and examined floral development with a focus on gynoecial development and ovule formation. Our analyses of *seu pan* and *pan ant* double mutants indeed support the tenet that *PAN* plays a functional role during gynoecial and ovule development that can be revealed when either the activity of *SEU* or of *ANT* is compromised. We also report that *SEU* plays a previously unanticipated role in floral meristem termination. This is revealed by altered patterns of *WUS* expression and the strong enhancement of the *PAN* indeterminacy phenotype in the *seu pan* double mutants, particularly under short-day conditions. Additionally, our data suggests that *PAN* can act as a repressor of *AG* within sepals, in contrast to previous work indicating a role for *PAN* in the activation of *AG* in whorl 4. Our data suggest that both *PAN* and *SEU* have whorl-specific functions during the regulation of *AG* that are critical for generating the Arabidopsis flower. Furthermore, the role of *PAN* and *SEU* during both floral meristem termination and CMM development suggest a possible link between these developmental events.

## Materials and methods

### Plant material and growth conditions

Plants were grown under long-day conditions of 16 h of light or under short-day conditions of 8 h of light. Temperature in the growth chambers were kept between 22 and 26°C, however the temperature experienced by the plants is lower when the lights are off, thus short-day grown plants may be grown at a slightly lower average temperature than long-day grown plants. The *ant-1* and *seu-3* alleles were previously characterized (Klucher et al., 1996; Pfluger and Zambryski, 2004). The *pan* alleles used are SALK_031380, SAIL_247, and SALK_057190 with T-DNA insertions in the 5′UTR, 7th intron and the 3rd intron, respectively, (McElver et al., 2001; Sessions et al., 2002; Alonso et al., 2003). PCR was used to confirm genotypes (Table 1). The *pan 057190* (SALK_057190) allele was previously characterized as a RNA null allele via *in situ* hybridization to inflorescence and floral tissues (Maier et al., 2009, 2011). Plants for rosette leaf counts were grown under short-day conditions until after bolting. Rosette leaves were removed, with care to only count rosette leaves and not axillary, cauline, or cotyledon leaves.

**Table 1:** **Genotyping primers**.

**Allele name**	**Genotyping Oligos FW (5′–3′)**	**Genotyping Oligo RV (5′–3′)**	**Genotyping Oligo internal**	**Size of Fragments (in base pairs)**
*ant-1*	TTCCCTCAAACCAGAAACCA	GGGCTCATGGATAAGCTCAG	N/A	Wt: 131 bp Mutant: 109
*seu-3*	GAATTTGCTGCGGTTCCAACT	GAAAATGTTCCGCCTTCGAT	Restrict with *Bsl1*	Wt: 235 and 345 Mutant: 580
*pan 031380 (in 5′UTR)*	CGGTAACACACATGACACATATG	ATGGTGAAAACCATTGACTGG	LbB1	Wt: 1228 Mutant: 550
*pan SAIL_247 (in 7th intron)*	TTGCCTCAATAAATCAGCCTG	GAATTCTTGGCAGACACTTCG	pCSA110LB2	Wt: 1138 Mutant: 500
*pan 057190 (in 3rd intron)*	ACATCAACACGGCCAAGTAAC	TCTCTCCTCACTCCCTCCTTC	LbB1	Wt: 1219 Mutant: 650

The oligonucleotides and size of the PCR fragments from genotyping for each of the alleles we utilized in this paper.

### *in situ* hybridization

The protocol for *in situ* hybridization was described previously (Wynn et al., 2011). A more detailed protocol is located at http://www4.ncsu.edu/~rgfranks/research/protocols.html. The *AG* antisense probe was *in vitro* transcribed using the T7 promoter from the pCIT565 plasmid linearized with HindIII. The *AG* antisense probe generated is complementary to the *AG* cDNA sequence from +140 (relative to the ATG) through the 3′ end of cDNA. The antisense *WUS* probe is complementary to the entire *WUS* cDNA clone and is derived from BamHI cut pMHwus16 plasmid, a gift of Jenn Fletcher. The *AG* sense strand control probe was generated from a linearized pCIT565 plasmid (cut with XhoI) using the Sp6 promoter and contains the full length cDNA sequences. To generate the *PAN* antisense probe, the plasmid G50929 (ABRC) a full-length sequence-confirmed ORF/cDNA clone (Yamada et al., 2003) was cut with SalI to linearize and then the antisense probe was generated using the T7 polymerase. Due to the position of the SalI site, the *PAN* antisense probe generated is complementary to sequences from +746 through +1353 relative to the ATG start codon in the *PAN* cDNA.

### Tissue fixing and clearing

Tissue was fixed in 9 parts ethanol:1 part acetic acid for 2 h, then washed in 90% ethanol twice. Gynoecia were hand-dissected in ethanol and then moved into Hoyer's solution (70% ethanol, 5% gum arabic, 4% glycerol) for clearing and mounting on slides for visualization. Slides were examined with an Axioscop2 microscope (Zeiss) with Nomarski optics. Ovule counts were made from stages 11–14 gynoecia fixed on slides. Analysis of carpel bending and splitting was done under dissecting scope. Gynoecia were rated from 1 to 4 independently for bending as well as splitting. A severity score was given based on the following scoring system: 1, no defect; 2, mild defect; 3, moderate defect; 4, severe defect. All gynoecia were scored by the same individual, at the same time without knowledge of the genotype. All photos were captured with Q Capture software on a 5.0 RTV digital camera (Q Imaging, Surrey, BC, Canada). Data analysis was conducted in JMP Pro 10 (SAS Institute Incorporated, Cary, NC, USA) using multiple pair-wise comparison of the means with a Tukey-Kramer HSD test at an alpha of 0.05 or with a Student's *T*-test.

## Results

### *pan* mutant alleles condition enhanced reduction of ovule number in *seu* and *ant* mutant backgrounds

**Figure 1:**
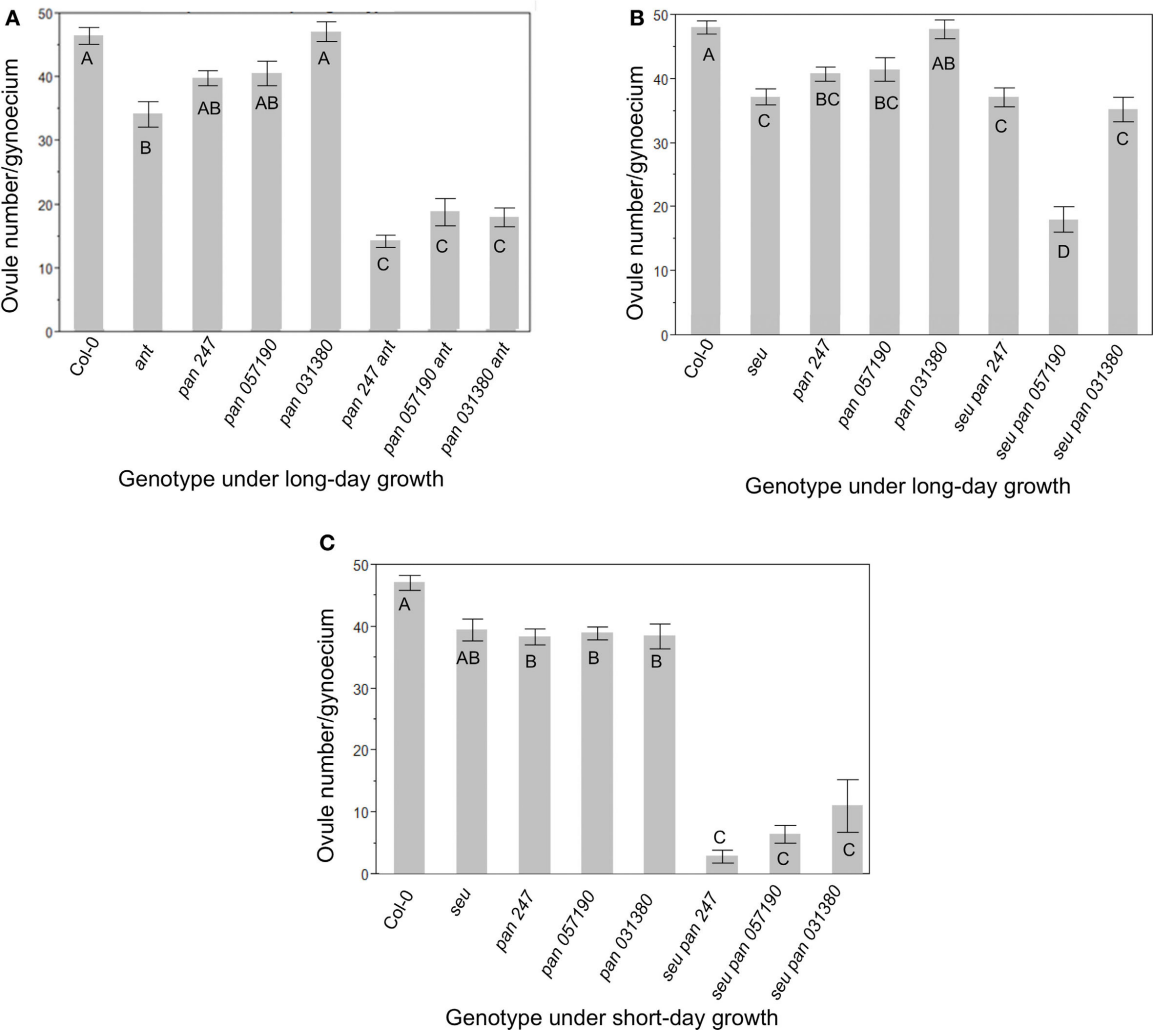
**Ovule number is decreased in *pan ant* and *seu pan* double mutants**. **(A)** Under long-day growth conditions *pan ant* double mutants displayed an enhanced loss of ovules relative to single mutant parents for all three *pan* alleles examined. **(B)** Under long-day growth conditions only the *seu pan 057190* plants showed a statistical reduction in ovule number compared to single mutants parents. **(C)** Under short-day growth conditions, the *seu pan* double mutants displayed an enhanced loss of ovules relative to single mutant parents for all three *pan* alleles examined. Comparisons for statistical differences across genotypes were made via pair-wise mean testing and the Tukey HSD *post-hoc* test—different letters indicate statistically different categories. Each error bar is constructed using 1 standard error from the mean.

In order to assay gynoecial development in *pan* mutant plants we characterized three available *pan* alleles (See Materials and Methods, Table 1) (McElver et al., 2001; Sessions et al., 2002; Alonso et al., 2003). Under our long-day growth conditions Col-0 plants averaged 46.9 ± 5.9 ovules per gynoecium. As has been previously reported, *ant* single mutants displayed significantly fewer (35 ± 8.7) ovules per gynoecium (Figure 1A) (Elliott et al., 1996; Klucher et al., 1996; Azhakanandam et al., 2008). Previously published characterizations of *pan* mutants did not report a reduction in ovule formation. We counted ovule primordia in the gynoecia of three different *pan* mutant alleles (Figure 1A). Although we detected slight reductions in ovule number in two out of three of the *pan* alleles we tested, these differences were not statistically different from Col-0. However, all three of these *pan* mutant alleles conditioned an enhancement of ovule loss in the *ant* mutant background (Figure 1A). Thus, in the *ant* mutant background *PAN* appears to provide an activity that supports ovule formation.

In a second set of experiments, we examined the function of *PAN* in the *seu* mutant background by assaying *seu pan* double mutants and the single mutant parents (Figure 1B). We again detected a slight reduction in ovule number in the *pan* single mutants relative to wild type, however this time the reduction was statistically significant in both the *pan 057190* and the *pan SAIL_247* alleles. The *seu* single mutant also conditioned a significant loss of ovules relative to Col-0. Furthermore, ovule loss in the *seu pan* double mutant was significantly enhanced by one of the three *pan* alleles (*057190*) that we tested under our long-day growth conditions (Figure 1B).

Short-day growing conditions have been previously shown to enhance the severity of mutant phenotypes of *pan* mutants (Maier et al., 2009, 2011). Under short-day conditions all three *pan* alleles displayed a statistically-significant reduction in ovule number relative to Col-0 grown under similar conditions (Figure 1C). Furthermore, all three alleles of the *seu pan* double mutant gynoecia exhibited an enhanced loss of ovules relative to either the *pan* or the *seu* single mutants. Thus, under the short-day growing conditions *pan* single mutants displayed a modest but significant reduction in ovule number relative to wild type, while ovule loss was enhanced in *seu pan* double mutants relative to the single mutants.

### *pan* mutant alleles condition enhanced disruptions of gynoecial morphology in *seu* and *ant* mutant backgrounds

**Figure 2:**
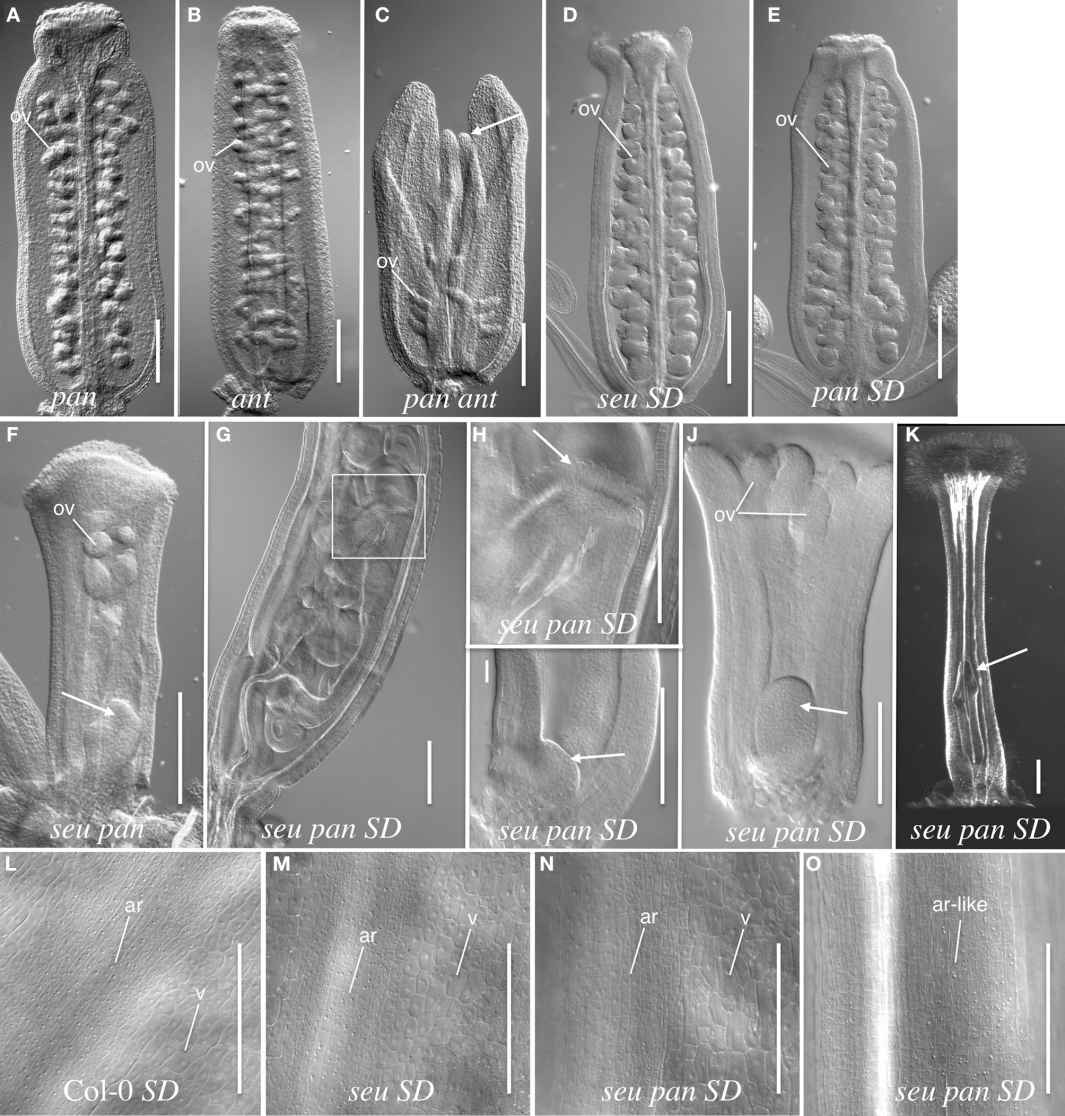
**Gynoecial and ovule phenotypes in *pan ant* and *seu pan* double mutants**. **(A)** *pan 057190* single mutant gynoecium. **(B)** *ant* single mutant gynoecium. **(C)** *pan 057190 ant* double mutant gynoecium displays reduced ovule number, a reduction in the growth of the medial domain (arrow) and enhanced splitting of the gynoecium. Arrow in panel **(C)** indicates the apical extent of the medial domain of the carpel. **(D)** *seu* single mutant gynoecium grown under short-day (SD) conditions shows slight splitting of the gynoecial apex. **(E)** SD-grown *pan* single mutant displays near wild type phenotype. **(F)** *seu pan 057190* double mutant (long-day conditions) displays a “fifth whorl” structure inside the gynoecium (arrow). **(G)** SD-grown *seu pan 057190* double mutant displays a well-developed “fifth whorl” structure inside the gynoecium. **(H)** A higher magnification image of the boxed area shown in panel **(G)**. Arrow indicates the stigmatic tissue at the apex of second gynoecium developing within the primary gynoecium. **(I)** SD-grown *seu pan* 057190 double mutant (stage 8) arrow indicates early stage of fifth whorl structure. **(J)** SD-grown *seu pan* 057190 double mutant (stage 9) arrow indicates fifth whorl structure. **(K)** SD-grown *seu pan 057190* double mutant displays a complete loss of ovule primordia, the loss of normal external valve cell surface morphology, and the presence of a “fifth whorl” structure (arrow). **(L–O)** epidermal cell morphology of external (abaxial) surface of the gynoecium. Distinctive cell surface morphology is observed in valve (v) and abaxial replum (ar) regions in Col-0 wild type **(L)**, *seu* single mutant **(M)** and a subset of *seu pan* double mutants **(N)**. However, in severely disrupted *seu pan* double mutants **(O)** the cell surface morphology of valve cells is not observed and all cells display an abaxial replum-like (ar-like) morphology. Scale bars in all panels are 200 microns, except for panels **(H–J)** where scale bars are 100 microns. ov, ovule; SD, Short-day growth conditions; ar, abaxial replum; v, valve.

The wild type Arabidopsis gynoecium is composed of two carpels that are fused along the carpel margins. The fused margins of the carpels are situated within the medial portion of the gynoecium. The growth of the medial gynoecial domain is reduced in the *seu ant* mutant resulting in gynoecial splitting and a loss of ovule formation (Azhakanandam et al., 2008). Thus, we examined the *pan* single and *seu pan* and *pan ant* double mutant gynoecia for gynoecial splitting and other gross morphology disruptions. Alterations to the overall gynoecial morphology were evident under both long- and short-day conditions, but they were more pronounced in plants grown under short-day conditions (Figure 2).

**Figure 3:**
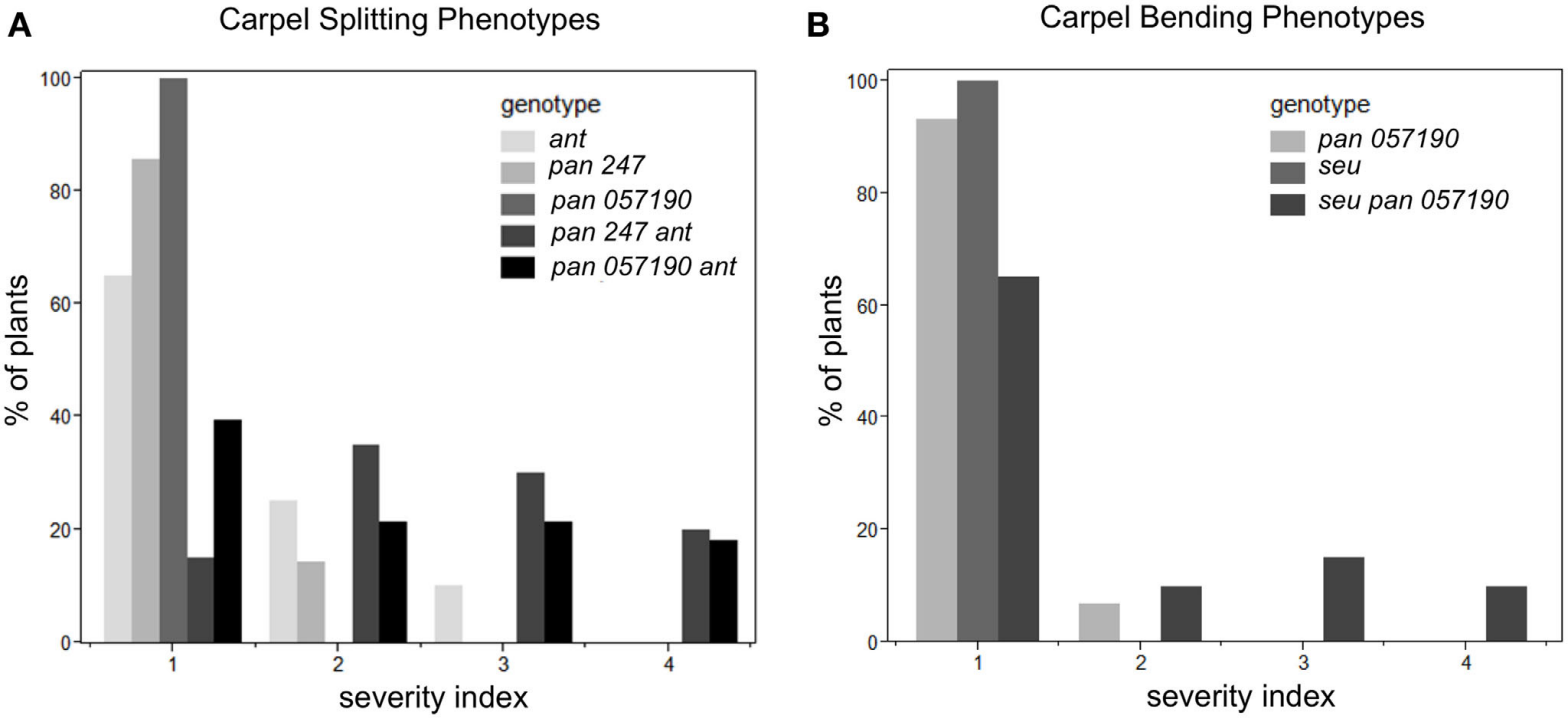
**Enhanced severity of carpel splitting in *pan ant* and carpel bending in *seu pan* double mutants under long-day growth conditions**. **(A)** The *pan ant* double mutants showed a statistically more severe carpel splitting than the single mutant parents. **(B)** *seu pan* double mutant plants showed statistically more severe carpel bending than the single mutant parents. Severity index for the carpel splitting: 1, no splitting; 2, mild splitting (flared tips or slight split); 3, moderate splitting (highly flared); 4, severe or completely split. Severity index for carpel bending: 1, no bending; 2, slight bending; 3, moderate bending; 4, severe bending. Statistical analysis was conducted with a Student's *T*-test and *p*-values less than 0.05.

Under long-day growth conditions, we evaluated gynoecia for both carpel splitting and carpel bending phenotypes employing a severity index from 1 to 4 (See Materials and Methods). We then used this severity index to generate a mean severity score for the comparison of genotypes of interest. Carpel bending and carpel splitting phenotypes were not observed in the Col-0 gynoecia that we assayed. Under the long-day growth conditions, the *ant* single and *pan* single mutants did display a mild degree of carpel splitting (Figure 3A). However, the *pan ant* double mutants displayed a statistically significant enhancement of carpel splitting compared to the single mutant gynoecia (Figures 2C, 3A). This is manifested by a greater proportion of double mutant gynoecia for which splitting was characterized as moderate or severe. Although *seu* single mutants displayed a mild degree of carpel splitting (Figure 2D), the carpel splitting phenotype was not enhanced in the *seu pan* double mutant under long-day conditions (Figure 2F).

Using a similar severity index we evaluated carpel bending. The *seu pan 057190* showed statistically significant enhancement in carpel bending compared to the *pan* single mutant (*seu* single mutants rarely display a bending phenotype under long-day) (Figure 3B). The bending phenotype appears to be the result of the gynoecium consisting of only one carpel that fuses to itself (data not shown). These data suggest that *PAN* and *SEU* play a role in both promotion of medial domain development and in the proper formation of two carpels in whorl four.

Under short-day growing conditions the severity and penetrance of *seu pan* gynoecial defects were enhanced relative to the long-day conditions (Figures 2G–O). (We did not examine the development of the *pan ant* double mutants under the short-day growing conditions.) To analyze the *seu pan* phenotypes under short-day conditions we scored the gynoecia for the occurrence of four phenotypes; complete loss of ovules, carpel bending, severe loss of valves (based on external cell-type morphology), and indeterminate growth from internal gynoecial positions. We did not observe any of these phenotypes in the Col-0 plants and they were found infrequently (5%) in *seu* or *pan* single mutants (Table 2). The *seu pan* double mutants, however, frequently displayed severe ovule loss, loss of the external valve tissue morphology, carpel bending, and/or indeterminacy phenotypes (Figures 2G–K; Table 2). In the Col-0 and single mutant gynoecia the surface morphology of the abaxial replum (ar) cells is distinct from that of the valve (v) cells (Figures 2L–M). Thus, these cell fates can be distinguished by the external cell surface morphology. In a subset of the *seu pan* gynoecia from short-day grown plants, the cells of the valve and abaxial replum could still be distinguished (Figure 2N). However, between 33 and 63% (depending on the mutant *pan* allele) of the *seu pan* double mutants displayed a severe alteration in the morphology of the external valve cells (Figure 2O). In these gynoecia we could not identify cells with the surface morphology that is indicative of valve cell identity. Instead all of the external gynoecial cells appeared to resemble abaxial replum cells (ar-like in Figure 2O).

**Table 2:** **Gynoecial disruption in short-day grown plants**.

**Genotype**	**Complete loss of ovules (%)**	**Carpel bending (%)**	**Loss of external valve tissue morphology (%)**	**Fifth whorl structure within the carpels (%)**	**Number of gynoecia counted**
Col-0	-	-	-	-	18
*Seu*	-	5	-	-	20
*pan 057190*	-	-	-	5	21
*pan 247*	-	-	-	5	20
*seu pan 051790*	35	33	33	71	31
*seu pan 247*	52	63	63	32	19

Categorization of the significant gynoecial disruptions. Note: phenotypes are not mutually exclusive and thus percentages can add up to more than 100%.

Other phenotypes observed in the *seu pan* double mutant under short-day growth conditions included five sepals, a reduction in petal size and number (typically two reduced petals per flower) and reduced production of pollen from anthers (data not shown). Additionally, the *seu pan* double mutant plants displayed an enhanced delay in the transition from vegetative to reproductive development as determined by counting the number of rosette leaves formed before bolting (Figure S1).

### Floral meristem indeterminacy is enhanced in the *seu pan* double mutants under short-day growing conditions

In the wild type Arabidopsis flower, the floral meristem terminates after the formation of the gynoecium. *AG* is required to promote the termination of the floral meristem (floral determinacy) by repressing the expression of *WUS*, a stem cell maintenance gene within the floral meristem (Laux et al., 1996; Mayer et al., 1998; Parcy et al., 1998; Busch et al., 1999; Lohmann et al., 2001; Lenhard et al., 2002). We examined *seu pan* double mutants to determine the extent of floral indeterminacy under both long-day and short-day conditions. We characterized fifth whorl structures as an over-proliferation of cells at the base of the gynoecium (Figures 2F–K). These structures were typically enclosed within the gynoecial tube.

These fifth whorl structures were observed in 15% of *seu pan 057190* and *seu pan 247* double mutants in long-day tissues, but not seen in either single mutant. Under short-day conditions the presence of fifth whorl structures was significantly more frequent in *seu pan* plants compared to both the frequency in the single mutants as well as to the double mutants grown in long-day conditions (Table 2). The fifth whorl structures appeared larger and more elaborated under short-day conditions. This data suggests that both *PAN* and *SEU* function to promote floral meristem determinacy and that this phenotype is more penetrant under the short-day growing conditions.

### *WUS* expression and inflorescence meristem structure are altered in *seu pan* double mutant plants

**Figure 4:**
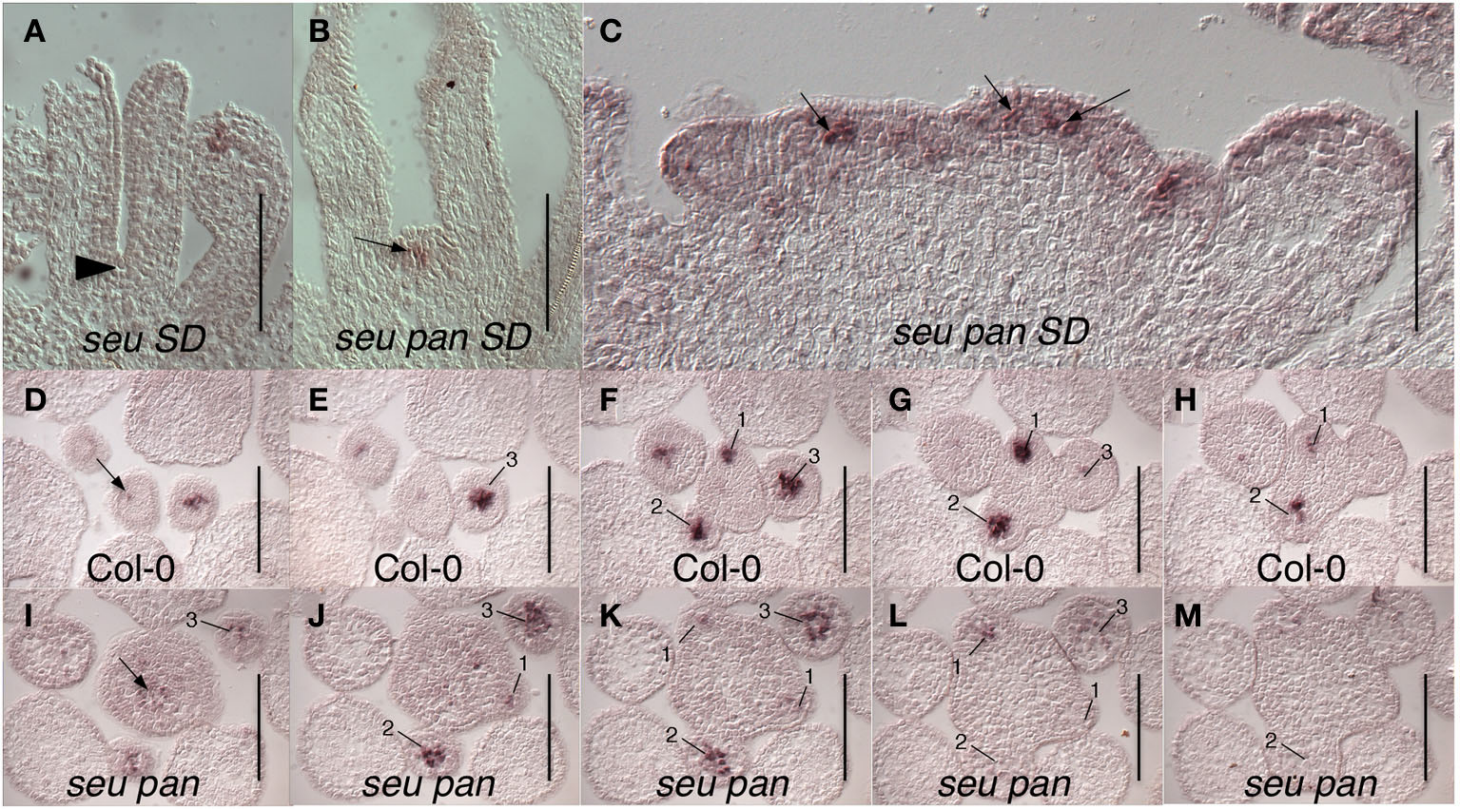
***WUS* expression in *seu pan* double mutants**. **(A)** No *WUS* expression is detected at the base of the stage 8 gynoecium (arrowhead) in this longitudinal section of a Col-0 flower. **(B)** *WUS* expression is detected in fifth whorl structure (arrow) at the base of the stage 8 *seu pan* gynoecium. **(C)** Longitudinal section of a fasciated inflorescence meristem (IFM) from *seu pan* double mutant where *WUS* expression appears in multiple foci (arrows) as well as diffusely throughout IFM. **(D–M)** Serial cross sections through Col-0 **(D–H)** and *seu pan* **(I–M)** inflorescences. Within a given genotype each cross section is 8 microns below the preceding cross section. **(D–H)** WUS expression is detected in the organizing center of the IFM (arrow) and in central zones of stage 1–3 floral meristems (numbered). **(I–M)** In the *seu pan* double mutants, the region of *WUS* expression appears more diffuse and somewhat expanded, both in the INF (arrow) and in the developing floral meristems (numbered). Note also that the IFM is larger in the *seu pan* double mutant. Scale bars are 100 microns in all panels.

The fifth whorl structures formed in the *seu pan* double mutants gynoecia were also examined for *WUS* expression as a marker for indeterminacy and a persistent functioning meristem. We occasionally were able to detect ectopic *WUS* expression in the fifth whorl structures (Figure 4B) suggesting that ectopic WUS expression may contribute to the formation of fifth whorl structures. However, we found additional examples of fifth whorl structures that did not express *WUS* (data not shown). Thus, although we were able to document cases of perdurant *WUS* expression, our data suggests that this ectopic expression is likely relatively short in duration and that *WUS* expression is not continuously maintained in the fifth whorl structures.

We also detected *WUS* expression in the center of the shoot apical meristem (SAM) and in early stage floral primordia (Figure 4). In Col-0, *WUS* is tightly expressed in a small number of cells in both the SAM and floral meristem (Laux et al., 1996; Mayer et al., 1998) (Figures 4D–H). In the *seu pan* mutant, the domain of *WUS* expression within both the SAM and the floral meristem appeared broader and more diffuse than it was in the wild type samples (Figures 4I–M). Although we did not quantify the size of the IFM, or observations of *in situ* sections showed instances where the size of the *seu pan* IFM was enlarged relative to the Col-0 or single mutant parents (compare 4D to 4I). In some of the *seu pan* inflorescence meristems the *WUS* expressing region was expanded and appeared to be punctuated as if several organizing centers had been formed within the potentially compound inflorescence meristem (Figure 4C; arrows). Thus, the disruption of *WUS* expression or accumulation in the IFM and early stage floral meristems as well as an ectopic persistence of expression within the floral meristem may contribute to the morphological disruptions observed in the *seu pan* mutant flowers.

### *AGAMOUS* is mis-expressed in whorl one of the *seu pan* double mutant flowers

In light of the known role of *AG* in regulating floral determinacy, and *SEU* and *PAN* acting as regulators of *AG* expression, we examined *AG* expression patterns in *seu pan* plants via *in situ* hybridization. A dominant negatively-acting *PAN-RD* transgene, in which a transcriptional repression domain has been fused to the PAN coding sequences, has been shown to condition floral indeterminacy that was correlated with a reduction of *AG* expression within whorl four (Das et al., 2009). We also sought to determine if the levels of *AG* were reduced in whorl four in the *pan seu* double mutants. However, we could not detect a consistent reduction in the levels of *AG* expression in whorl 4 under long-day or short-day growth conditions.

**Figure 5:**
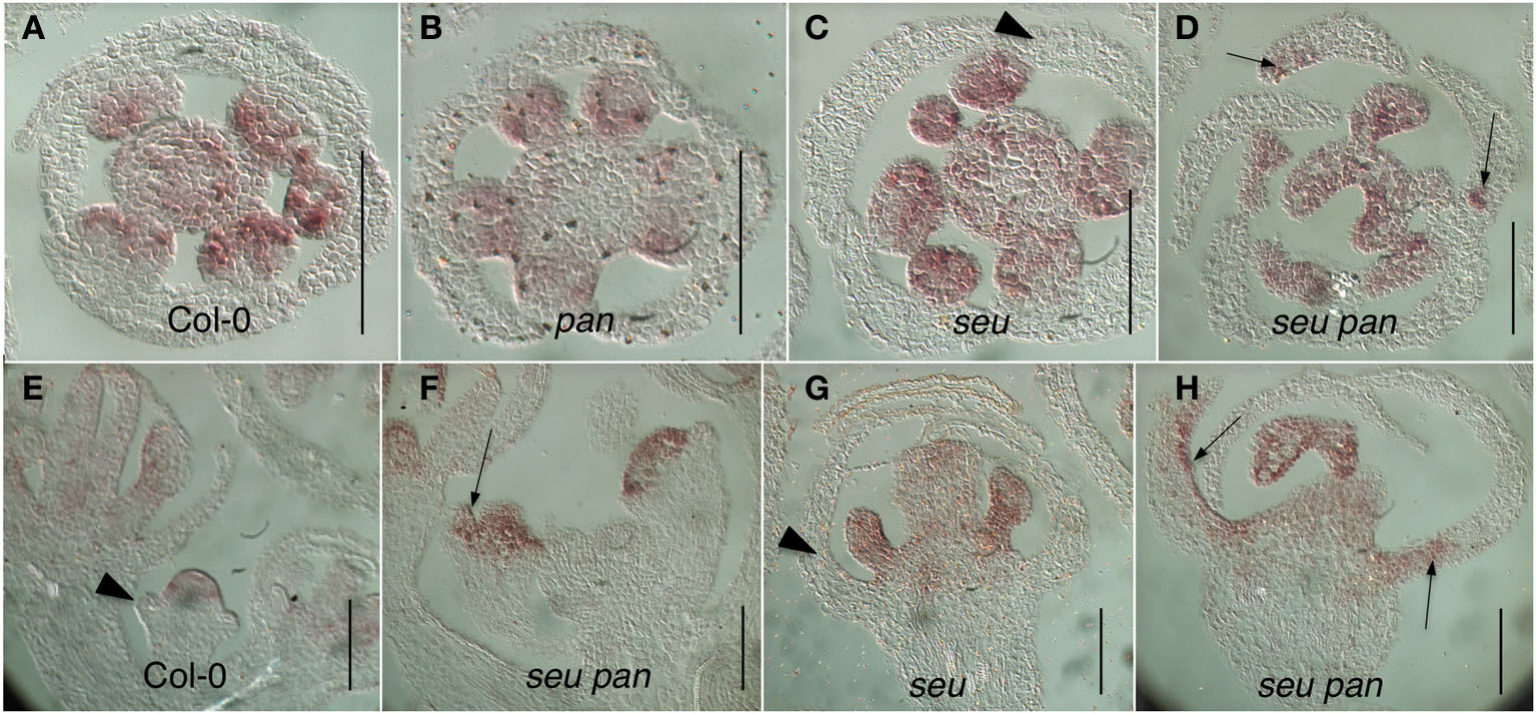
**Ectopic *AG* expression in the *seu pan* double mutants**. **(A–D)** Floral cross sections **(E–H)** Floral longitudinal sections. Arrowheads indicate sepals within which *AG* expression is not detected. Arrows indicate sepals within which ectopic *AG* expression is detected. Ectopic expression of *AG* is most strongly detected in the adaxial and marginal portions of the developing first whorl organs in the *seu pan 057190* double mutants (panels **D,F,H**). Scale bars are 40 microns in length.

**Figure 6:**
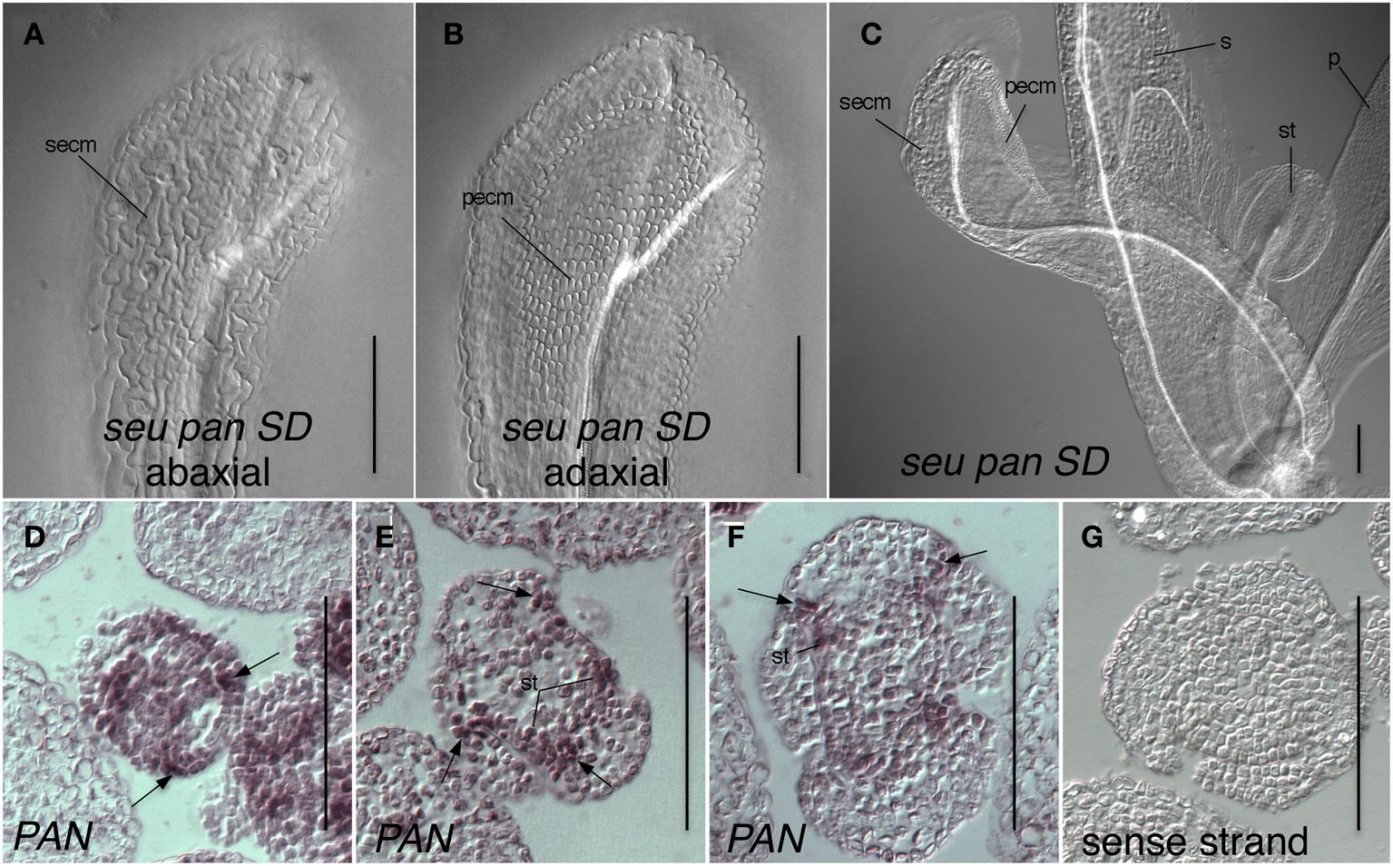
**Expression of *PAN* in adaxial portions of sepals is consistent with a role for *PAN* in the repression of *AG* and specification of organ identity**. **(A–C)** Differential interference contrast optics microscopic image of chloral hydrate cleared tissue. **(A)** Abaxial surface of whorl one organ in *seu pan* short-day (SD)-grown flower displays cells with sepal epidermal cell morphology (secm). **(B)** Adaxial surface of same organ as in panel **(A)** displays petal epidermal cell morphology (pecm). **(C)** A *seu pan* flower displays a partial homeotic transformation of whorl one organ on adaxial surface as indicated by the presence of petal epidermal cell morphology (pecm). Morphologically normal petal (p), sepal (s), and stamen (st) are indicated. **(D)** Stage 3 floral cross section. *PAN* expression is detected in adaxial and marginal portions of developing sepals (arrows) as well as in stamen anlagen and floral meristem. **(E,F)** *PAN* expression is detected in adaxial and marginal portions of developing sepals (arrows) as well as in stamen primordia (st) in stage 5 **(E)** and stage 6 **(F)** floral cross sections. **(G)** sense strand control hybridization on stage 5 floral cross section. Scale bars are 100 microns in all panels.

Somewhat unexpectedly, we frequently observed instances of ectopic *AG* expression in *seu pan* double mutant whorl 1 organs (Figures 5D,F,H). Upon closer examination of the external cell morphology of floral organs from the *seu pan* flowers, we detected instances of chimeric organs in whorls one including partially petaloid and stamenoid organs (Figure 6). These partial homeotic organ transformations have been reported in the *seu* single mutant previously (Franks et al., 2002) but are rarely seen in the *seu-3* allele in the Col-0 background that we have used in this study (Pfluger and Zambryski, 2004). We found the 30% (*N* = 43) of the *seu pan* whorl one floral organs exhibited partial homeotic transformations based on the cell surface morphologies (Table 3). These homeotic transformations were not observed in the *seu* and *pan* single mutant parents under our growth conditions. Based on the organ type specific cell surface morphology, the *seu pan* whorl one organs appeared to be sepals that were partially converted to petals (Figure 6). The presence of cells with the classic petal cell morphology was most often observed on the adaxial and marginal portions of the sepals (Figures 6A–C). These data are consistent with *PAN* acting in a partially redundant fashion with *SEU* during the repression of *AG* in the developing sepals. As previous accounts of *PAN* expression (Chuang et al., 1999) did not report *PAN* expression in whorl one organs, we examined expression of *PAN* via *in situ* hybridization to look carefully at the developing whorl one organs. We detected expression of *PAN* in portions of the developing whorl one primordia in wild type floral buds during stages 3–6. This was chiefly confined to the adaxial and marginal portions of the developing sepals (Figures 6D–F). Thus, there is a good correlation between the expression domain of the *PAN* transcript in wild type and the presence of homeotic cell type transformation in the *seu pan* mutant.

**Table 3:** **Chimeric floral organs in *seu pan* double mutants under short-day conditions**.

**Genotype**	**% Chimeric sepals**	**% Chimeric petals**
Col-0	0 (*N* = 19)	0 (*N* = 8)
*seu-3*	0 (*N* = 32)	0 (*N* = 26)
*pan 057190*	0 (*N* = 23)	0 (*N* = 11)
*seu-3 pan 057190*	30 (*N* = 43)	0 (*N* = 11)

## Discussion

### Role of *PAN* in gynoecial medial domain development

The enhancement of gynoecial defects observed in the *seu pan* and *pan ant* double mutants relative to the single mutant parents indicates (1) that *PAN* plays a role in the development of the medial domain of the gynoecium and that (2) this role is revealed when the activity of *SEU* or *ANT* is compromised. The dependence of the phenotype on the loss of function of *SEU* or *ANT* suggests that the function of *PAN* during gynoecial development is partially overlapping with that of*SEU* and *ANT*. The defects of the *seu pan* double mutant are similar to, but distinct from the *ant pan* double mutant, indicating a differential sharing of functions between these three genes.

The defects observed in the *pan ant* double mutants include an enhanced loss of ovules and enhanced splitting of the gynoecial tube relative to the single mutant parents (Figures 1A, 3A). Both of these we interpret as the result of a reduced growth of the medial domain of the gynoecium. In the *pan ant* double mutants the medial domain does not grow to the same extent as the neighboring lateral domains (Figure 2C). This may result in the failure of the gynoecial tube to fuse completely, as well as contribute to a loss of ovule primordia.

The *seu pan* double mutant also displays an enhanced loss of ovules, although this is not as severe as that observed in the *pan ant* double mutant. Gynoecial splitting was not enhanced in the *seu pan* double mutants. The severity of carpel bending was enhanced in the *seu pan* double mutants relative to the parental genotypes. The enhanced curving of the gynoecial tube resulted from the loss of one of the two component carpels and the fusion of the remaining carpel upon itself (data not shown). This data suggests that *SEU* and *PAN* share a function in the regulation of carpel number. In the most severely affected *seu pan* double mutants, there is a loss of the characteristic morphology of the abaxial valve epidermal cells and these cells develop as cells that are morphologically similar to abaxial replum cells (Figures 2L–O) suggesting that patterning along the medial lateral extent of the gynoecium may be affected. The analysis of additional markers of the medial and lateral domains in the *seu pan* double mutant would help to determine if there is an alteration in medial/lateral patterning events in the *seu pan* double mutant.

*PAN* is expressed within the adaxial portions of the medial domain and in the developing ovule primordia (Chuang et al., 1999; Wynn et al., 2011) and thus may directly regulate genes within the medial domain that support medial domain development. Alternatively, as *PAN* is also expressed within the vegetative shoot apex, as well as the IFM and developing floral meristems (Chuang et al., 1999; Fulcher and Sablowski, 2009; Maier et al., 2009) (Figure 6), the effects of *PAN* on earlier stage floral meristems or perhaps the IFM may lead to later effects on the development of ovules from the medial domain.

Previous analyses of *pan ettin (ett)* and *pan tousled* (*tsl*) double mutants indicated that *PAN* also shares overlapping functional roles with *TSL* and *ETT* during medial domain development (Roe et al., 1997; Sessions et al., 1997). The carpels of the *pan tsl* double mutant gynoecia are completely unfused and serrated at their margins (Roe et al., 1997). The CMM-derived tissues are also significantly reduced in these gynoecia and very few ovules develop. Similarly Sessions et al. reported a synergistic loss of ovule and placental development in the *pan ett* double mutant (Sessions et al., 1997). Thus, a variety of non-additive genetic interactions affecting gynoecial development have been described for the *seu*, *lug*, *pan*, *ant*, *ett*, and *tsl* higher order mutants (Roe et al., 1997; Sessions et al., 1997; Liu et al., 2000; Franks et al., 2002; Pfluger and Zambryski, 2004; Azhakanandam et al., 2008).

### Role of *SEU* and *PAN* in floral meristem determinacy

*PAN* has been previously shown to function in the termination of the floral meristem and to function as an activator of *AG* (Das et al., 2009; Maier et al., 2009). The direct binding of PAN to conserved regulatory elements within the *AG* second intron and the functional importance of these elements in generating the *AG* expression pattern strongly suggests that PAN directly functions as an activator of *AG* expression. Consistent with this Maier et al. reported a reduction in *AG* transcript in the *pan* single mutant, but only when this mutant was grown under short-day conditions (Maier et al., 2009). Das et al. did not detect a reduction in the *pan* single mutant, but did observe a reduction of *AG* expression within whorl 4 in plants that carried a dominant negative *PAN-RD* construct in which *PAN* is fused to a strong transcriptional repressor domain (Das et al., 2009). These results suggest that the function of redundant regulatory elements within the *AG* second intron (Sieburth and Meyerowitz, 1997; Bomblies et al., 1999; Deyholos and Sieburth, 2000) and of redundant bZIP family members (Das et al., 2009; Maier et al., 2009) may reduce the phenotypic consequences of the loss of *PAN* function on *AG* expression. Das et al. and Maier et al. both reported ectopic expression of *WUS* in fifth whorl structures in *pan* single mutants. However, they did not report alterations to *WUS* expression patterns at earlier stages of floral development. We also did not observe altered *WUS* expression in *pan* single mutants. However, we observed alterations of the *WUS* expression patterns that are evident in the *seu pan* double mutant, particularly when grown under short-day conditions. In these cases *WUS* expression was often more diffusely localized within the IFM and the developing floral meristems. Additionally we observed instances of perdurant *WUS* expression within the developing fifth whorl structures. Thus, a deregulation of *WUS* expression or localization is likely to contribute to the indeterminacy phenotypes observed in the *seu pan* double mutants.

As *PAN* functions as an activator of *AG* transcription, and *AG* as a repressor of *WUS* expression, this deregulation of *WUS* may be caused by a reduction in *AG* transcription or by post-transcriptional regulation of *AG* activity or both. We did not detect a consistent reduction in the levels of *AG* expression in either the *pan* single mutant or the *seu pan* double mutant under either long-day or short-day growth conditions. Yet the fifth whorl indeterminacy phenotypes were clearly enhanced in the *seu pan* double mutant under the short-day growing conditions. It is possible that the *in situ* hybridization assay is not sensitive enough to detect modest, yet biologically-significant reductions in *AG* transcript. Alternatively, as SEU can physically interact with several MADS domain-containing proteins that dimerize with AG (e.g., AP1, SEP3, SVP, and AGL24) (Gregis et al., 2006; Sridhar et al., 2006; Smaczniak et al., 2012), we propose that the SEU protein may regulate the ability of AG to function via physical interactions with these MADS domain proteins. Thus, the ability of the AG protein to repress *WUS* expression and thus bring about floral stem cell termination may be compromised in the *seu* mutant background. This would be consistent with the ectopic persistence of *WUS* expression observed in the *seu pan* fifth whorl structures. It also might contribute to the fasciation defects we observed and to the expansion of the *WUS* expression domain in the IFM and floral meristems.

### Whorl specific action of *PAN* and *SEU* in the repression of *AG* expression and specification of organ identity

*SEU* functions as a transcriptional adaptor required for the repression of *AG* transcription within whorls 1 and 2 (Franks et al., 2002; Gregis et al., 2006; Sridhar et al., 2006; Gonzalez et al., 2007). SEU forms a complex with several MADS domain-containing proteins and the transcriptional co-repressor LUG to bind to the second intron sequences of *AG*. This brings about repression via histone deacetlyation (Sridhar et al., 2006; Gonzalez et al., 2007). The loss of *PAN* activity in the *seu pan* double mutant enhances the de-repression of *AG* in whorl 1 structures and leads to the partial homeotic transformation of sepals (Figures 5, 6). Thus, we suggest that *PAN* functions in the repression of *AG* in whorl 1, a function that is partially overlapping with *SEU*. As we have detected *PAN* transcript in the adaxial and marginal portions of the developing sepals, a direct role for *PAN* in the repression of *AG* is plausible.

We have been unable to demonstrate a physical interaction between SEU and PAN in a yeast two hybrid assay (data not shown). However, both *PAN*, as well as SEU-containing complexes have been shown to bind directly to the DNA regulatory elements found within the *AG* second intron (Sridhar et al., 2004, 2006; Das et al., 2009; Maier et al., 2009). Thus, we favor a model in which both SEU and PAN via direct interaction with the *AG* second intron bring about the repression of *AG* expression within developing whorl one organs. The action of additional redundant regulators of *AG* repression (*AP2*, and *SEUSS-LIKE* family members) (See for review Liu and Mara, 2010) likely buffers the extent of *AG* de-repression that is observed in the *seu pan* double mutant. Furthermore, the mis-specification of petal cell identity in the whorl 1 structures suggests that B-class genes required for petal identity specification (e.g., *PI* and *AP3*) are also likely to be de-repressed in the *seu pan* double mutant, although we have not yet confirmed this with *in situ* hybridization experiments. Ectopic expression of B-class genes in whorl one organs could be caused by ectopic *AG* expression. Previously *lug* alleles were shown to condition the partial transformation of whorl one organs to petaloid and stamenoid chimeric organs and ectopic expressions of the B-class genes *PISTILLATA* (*PI*) and *APETALA3* (*AP3*) were detected in developing whorl one structures (Liu and Meyerowitz, 1995). Liu and Meyerowitz demonstrated that the petaloid characteristics of the whorl one organs in *lug* mutants were dependent on ectopic *AG* expression (i.e., the petaloid characteristics were not observed in the *lug ag* double mutants). They propose that the ectopic *AG* brings about an ectopic expression of the B-Class genes and this is consistent with the identification of *PI* and *AP3* as targets of *AG* regulation by Gomez-Mena et al. (2005).

Our data suggests that *SEU* and *PAN* function in a whorl specific fashion in the regulation of *AG* transcription or activity. *SEU* and *PAN* activities are required for efficient repression of *AG* in whorl 1 while their activities are required for efficient activation of *AG* function in whorl four. We propose that the differential action of these proteins is due to whorl specific co-factors or post-transcriptional modifications. The identity of these whorl specific modifiers remains to be elucidated.

### Relationship between floral meristem termination and CMM development

Zuniga-Mayo et al. previously suggested a relationship between the proper termination of the floral meristem and the subsequent development of the CMM (Zuniga-Mayo et al., 2012).

This was based on their analysis of *jaiba crabs claw* double mutants that display a loss of floral determinacy as well as defects in the development the CMM. Our investigation of the *seu pan* double mutant further supports this possibility. When ovules develop in the *seu pan* double mutants, they arise at apical positions within the gynoecium, and thus at a distance from the basally-located fifth whorl structures. Thus, if the floral meristem fails to properly terminate, the cells furthest from the perdurant meristem are more likely to form ovules than those closer to the meristem. This could suggest a gradient of an inhibitor from the floral meristem. However, it is equally likely that a temporal effect explains the difference. As the gynoecium grows from the apex, cells that divided temporally later in development will also be found in more apical positions. Thus, the decay over time of any inhibitory effect of the floral meristem might also result in the formation of ovules only at the apex of the *seu pan* double mutants.

## Author contributions

April N. Wynn and Robert G. Franks: conceived and designed the experiments, analyzed the data, and wrote the paper; April N. Wynn, Andrew A. Seaman, Ashley L. Jones and Robert G. Franks: Performed the experiments.

### Conflict of interest statement

The authors declare that the research was conducted in the absence of any commercial or financial relationships that could be construed as a potential conflict of interest.

## Supplementary material

Figure S1 **Short-day growth conditions reveal a floral transition delay in seu *pan 057190* double mutants** Time to floral transition (in number of rosette leaves produced) indicates that both the *seu* single mutants and the *seu pan* double mutants have a delay in the transition to flowering. The *seu pan* plants are significantly more delayed than the *seu* single mutants. Comparisons for statistical differences across genotypes were made via pair-wise mean testing and the Tukey HSD *post-hoc* test—different letters indicate statistically different categories. Each error bar is constructed using 1 standard error from the mean. *pan* mutant alleles enhance the floral transition delay observed in *seu* single mutants. When examining *seu pan* plants grown in short-day conditions it was apparent that the *seu pan* double mutant plants were slower to transition to the reproductive growth phase. To quantify this delay, we counted the number of rosette leaves produced before the plants transition to a reproductive growth phase. The reproductive growth phase is characterized by the formation of cauline leaves and internode elongation. Under the short-day growing conditions the Col-0 plants produce on average 40.8 ± 4.7 rosette leaves before transition to a reproductive growth phase (Figure S1). *pan* mutants show no statistically significant difference when compared to Col-0 (43.2 ± 4.5 leaves). In contrast *seu* mutants exhibit a statistically significant delay in flowering relative to Col-0, producing 50.5 ± 4.0 leaves prior to bolting. Mutations in *SEU* also caused a delay in the floral transition in long-day growth conditions (Wynn and Franks, unpublished). When we examined the *seu pan* double mutants we observed that they were significantly delayed, relative to the single mutants, producing on average 60.4 ± 5.1 leaves before the transition. These results suggest that both *PAN* and *SEU* function to promote the transition to reproductive growth phase under short-day growing conditions.
