# Corrigendum: [Novel functional roles for *PERIANTHIA* and *SEUSS* during floral organ identity specification, floral meristem termination and gynoecial development]

**DOI:** 10.3389/fpls.2014.00434

**Published:** 2014-08-29

**Authors:** Robert G. Franks

**Affiliations:** Department of Plant and Microbial Biology, North Carolina State University Raleigh, NC, USA

**Keywords:** corrigendum, hoyers, chloral hydrate, *PERIANTHIA*, *SEUSS*

In “Materials and Methods” under the heading “Tissue Fixing and Clearing” we incorrectly described the composition of our Hoyer's solution. “… Hoyer's solution (70% ethanol, 5% gum arabic, 4% glycerol)” should read “… Hoyer's solution (70% chloral hydrate, 5% gum arabic, 4% glycerol).”

## Conflict of interest statement

The author declares that the research was conducted in the absence of any commercial or financial relationships that could be construed as a potential conflict of interest.

