# Supplementary figures and images for: Novel functional roles for PERIANTHIA and SEUSS during floral organ identity specification, floral meristem termination, and gynoecial development
Source: Front Plant Sci. 2014 Apr 7;5:130. doi: 10.3389/fpls.2014.00130 (PMC3985007; doi:10.3389/fpls.2014.00130)

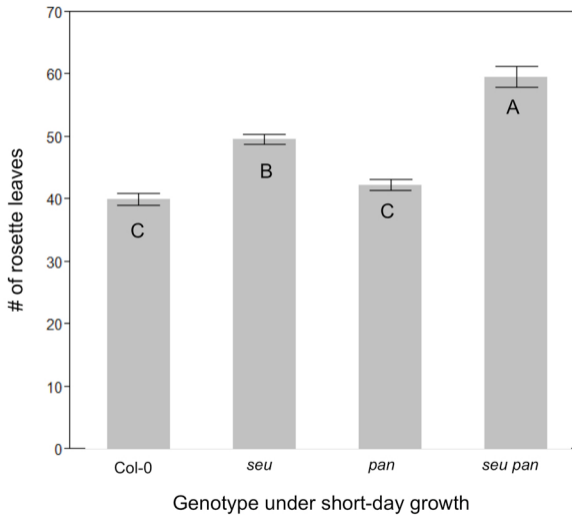

Supplement: Figure S1 — Short-day growth conditions reveal a floral transition delay in seu pan 057190 double mutants Time to floral transition (in number of rosette leaves produced) indicates that both the seu single mutants and the seu pan double mutants have a delay in the transition to flowering. The seu pan plants are significantly more delayed than the seu single mutants. Comparisons for statistical differences across genotypes were made via pair-wise mean testing and the Tukey HSD post-hoc test—different letters indicate statistically different categories. Each error bar is constructed using 1 standard error from the mean. pan mutant alleles enhance the floral transition delay observed in seu single mutants. When examining seu pan plants grown in short-day conditions it was apparent that the seu pan double mutant plants were slower to transition to the reproductive growth phase. To quantify this delay, we counted the number of rosette leaves produced before the plants transition to a reproductive growth phase. The reproductive growth phase is characterized by the formation of cauline leaves and internode elongation. Under the short-day growing conditions the Col-0 plants produce on average 40.8 ± 4.7 rosette leaves before transition to a reproductive growth phase (Figure S1). pan mutants show no statistically significant difference when compared to Col-0 (43.2 ± 4.5 leaves). In contrast seu mutants exhibit a statistically significant delay in flowering relative to Col-0, producing 50.5 ± 4.0 leaves prior to bolting. Mutations in SEU also caused a delay in the floral transition in long-day growth conditions (Wynn and Franks, unpublished). When we examined the seu pan double mutants we observed that they were significantly delayed, relative to the single mutants, producing on average 60.4 ± 5.1 leaves before the transition. These results suggest that both PAN and SEU function to promote the transition to reproductive growth phase under short-day growing conditions. [file Presentation1.PDF]
